# Potential impact of contaminated bronchoscopes on novel coronavirus disease (COVID-19) patients

**DOI:** 10.1017/ice.2020.102

**Published:** 2020-04-02

**Authors:** Cori L. Ofstead, Krystina M. Hopkins, Matthew J. Binnicker, Gregory A. Poland

**Affiliations:** 1 Ofstead and Associates, St Paul, Minnesota; 2 Mayo Clinic Department of Laboratory Medicine and Pathology, Mayo Clinic, Rochester, Minnesota; 3 Mayo Clinic Vaccine Research Group, Mayo Clinic, Rochester, Minnesota

*To the Editor—*During the novel coronavirus disease (COVID-19) pandemic, critically ill patients may require therapeutic bronchoscopy or sample collection via bronchoalveolar lavage (BAL), which involves using a bronchoscope to flush lungs with saline solution. Results of BAL assays are used to make clinical decisions that may impact outcomes. Clinicians have reported that COVID-19 patients had bacterial and fungal pulmonary coinfections^1^ with potential pathogens including *Escherichia, Salmonella, Pseudomonas*, and *Stenotrophomonas*.^2^ Recent research suggests that COVID-19 coinfections are associated with significantly higher mortality rates.^3^

Numerous nosocomial outbreaks and pseudo-outbreaks have been linked to inadequately reprocessed bronchoscopes.^4,5^ In 2018, researchers in Wuhan City, China, identified *Stenotrophomonas maltophilia* in 55.55% of BAL samples.^6^ The source was the channel of an improperly reprocessed bronchoscope, and the pseudo-outbreak involved 25 asymptomatic patients undergoing treatment for tuberculosis and other infections. Reprocessing and hand-hygiene deficiencies were identified.

Ofstead et al^7-9^ have conducted prospective studies that evaluated effectiveness of bronchoscope reprocessing in 5 hospitals in the United States. Microbial growth was detected on 23 of 35 bronchoscopes (65.7%), and 10 bronchoscopes (28.6%) harbored high-concern organisms or actionable levels of microbial growth (>100 CFU) (Table 1).^7-9^ Mold and gram-negative bacteria were detected, including *S. maltophilia, Sphingomonas phyllosphaerae*, and *Escherichia coli/Shigella*. At one hospital, high protein levels were detected in 7 of 8 bronchoscopes, indicating that manual cleaning failed to remove soil.^7^ Visual inspections using magnification and borescopes identified residue or defects in 100% of bronchoscopes.^7,8^ Audits evaluating personal protective equipment use and reprocessing guideline adherence (eg, point-of-care precleaning; leak testing; manual cleaning; visual inspection; cleaning verification; high-level disinfection; rinsing; drying; storage; transport and handling) identified breaches in all 5 hospitals.^7-9^ Technicians in 2 hospitals (sites 1 and 5) performed most reprocessing steps correctly, but bronchoscopes at both sites harbored *S. maltophilia* due to contaminated rinse water.^7,9^ In 3 hospitals (sites 2–4), nearly all steps were performed incorrectly or were skipped entirely.^7,8^ In light of these breaches and observations that most bronchoscopes were damaged and contaminated, a recommendation was made that procedures in 2 hospitals be halted until strict protocols could be implemented and personnel retrained. In addition, it was recommended that badly damaged bronchoscopes be removed from service and replaced with single-use, sterile bronchoscopes or new reusable bronchoscopes constructed with sterilizable materials.

**Table 1.** Microbial Culture Results From Fully Reprocessed Bronchoscopes in 5 Hospitals

Hospital ID	Scope	Model	After High-Level Disinfection			
			Surface CFU	Effluent CFU	Species Identification	Clinical Significance
Bronchoscope reprocessing effectiveness study^7^						
1	1	P190	0	3	*Kytococcus aerolatus*	Low concern
1	2	UC180F	0	0	NA	NA
1	3	P190	0	4	*Bacillus fastidiosus*; *B. litoralis*	Low concern
1	4	P190	0	0	NA	NA
1	5	P180	0	0	NA	NA
1	6	XP190	0	3	*Stenotrophomonas maltophilia*	High concern
1	7	P180	0	3	*S. maltophilia*	High concern
1	8	XP160F	0	0	NA	NA
1	9	3C160	0	0	NA	NA
1	10	P190	0	3	*Paenibacillus provencensis*	Low concern
2	11	UC180F	TNTC	88	*Sphingomonas phyllosphaerae*; *Escherichia coli/* *Shigella* spp; *Lecanicillium lecanii/* *Verticillium dahliae*; GPC	High concern, actionable growth level
2	12	1TH190	82	69	GPC	Actionable growth level
2	13	UC180F	22 (74)^a^	0	*E. coli/* *Shigella* spp; GPC	High concern
2	14	1TH190	36	163	GPC	Actionable growth level
2	15	UC180F	0	6	*S. maltophilia*; GPC	High concern
2	16	1TH190	72	0	GPC	Unknown
2	17	UC180F	0	0	NA	NA
2	18	1TH190	0	0	NA	NA
3	19	1TQ180F	0	0	NA	NA
3	20	UC180F	0	3	*Paenibacillus* spp	Low concern
3	21	1TQ180	0	0	NA	NA
3	22	1TQ180	4	0	*Staphylococcus epidermidis*; *Paenibacillus* spp	Low concern
3	23	1TQ180	0	3	*Paenibacillus* spp	Low concern
3	24	1TQ180	0	0	NA	NA
Endoscope drying effectiveness study^8^						
1	25	BF-P180	0	0	NA	NA
1	26	BF-P190	0	3	*Kocuria rosea*	Unknown
1	27	UC-180F	0	3	*S. epidermidis*; *Bacillus subterraneus*	Low concern
2	28	BF-1TH190	0	TNTC	*S. phyllsophaerae*; *B. lichenformis/* *B. cereus/* *B. sonorensis*	High concern; Actionable growth level
4	29	LF-2	0	18	*B. subtilis*	Low concern
4	30	LF-GP	0	6	*B. cereus*	Unknown
Microbial cultures toolkit study^9^						
5	31	BF-1TH190	0	0	NA	NA
5	32	BF-H190	1	1	*Delftia acidovorans*; *Rothia mucilaginosa*	Unknown
5	33	BF-H190	0	2	*S. maltophilia*	High concern
5	34	BF-H190	1	0	*S. epidermidis*	Low concern
5	35	BF-1TH190	1	0	*S. epidermidis*	Low concern

Note. CFU, colony-forming units; NA, not applicable; TNTC, too numerous to count; GPC, gram-positive cocci.

a Results from a swab of the ultrasound component of an EBUS bronchoscope appear in parentheses.

There is currently an urgent need to reduce the number of patients requiring hospitalization or intensive care, in part because of shortages of ventilators and personal protective equipment. Given the high bronchoscope contamination rates found during routine use in previous studies, we must now consider the possibility of bronchoscopy-associated transmission of COVID-19 or other pathogens that could cause secondary infections. Theoretically, high-level disinfection should eliminate these risks when bronchoscopes are well-maintained and reprocessed according to manufacturer instructions and professional guidelines. However, even during normal patient loads, practices are frequently substandard, and pathogens are commonly present on patient-ready endoscopes. The presence of gastrointestinal pathogens found in bronchoscopes and BAL samples suggests the possibility of cross-contamination caused by intermingling bronchoscopes and gastrointestinal endoscopes during reprocessing. This hypothesis is supported by findings at one hospital where protein and bioburden levels on brand-new bronchoscopes increased significantly following manual cleaning prior to any clinical use.^7^

Researchers recently reported COVID-19 patients presenting with diarrhea and abdominal pain, with fecal carriage of SARS-CoV-2 among severely ill and asymptomatic patients. Thus, extreme care must be taken to minimize cross-contamination during all endoscope reprocessing.

Reprocessing effectiveness has not been evaluated in epidemic settings, and research is needed to confirm that COVID-19, influenza viruses, and other pathogens are eliminated in these settings. The use of sterile, disposable bronchoscopes would substantially reduce the risks for patients and reprocessing personnel, and this approach has been recommended by the American Association for Bronchology and Interventional Pulmonology.^10^ However, single-use bronchoscopes are not universally available and may not be sufficient for advanced bronchoscopy. When reusable bronchoscopes must be used, they should be segregated from gastrointestinal endoscopes and sterilized rather than relying on high-level disinfection.

We urgently recommend further research assessing potential contamination of reusable bronchoscopes with viral, bacterial, and fungal pathogens. Laboratory methods should include bacterial/fungal cultures and molecular assays (eg, real-time PCR) for respiratory viruses, including COVID-19. To optimize the accuracy of results, samples should be taken from multiple components using a friction-based technique (eg, flush-brush-flush for sampling ports and channels). Laboratories should utilize methods that foster growth of microbes that are viable but not easily culturable (eg, using neutralizers to counteract residual reprocessing chemicals that could suppress growth, concentrating samples, and/or incubating for at least 5–7 days or 6–8 weeks when culturing for *Mycobacteria*). Due to the relative insensitivity of viral culture and potential safety concerns related to cultivating COVID-19, molecular testing (ie, targeted real-time PCR and multiplex respiratory panels) could be considered to assess contamination with viral pathogens.

No patient should suffer from preventable nosocomial infections due to bronchoscopy. Using bronchoscopes that have physical defects and harbor viruses, bacteria, or fungi puts vulnerable patients at risk and could have adverse effects on public health. Institutions are obligated to protect both patients and reprocessing personnel and ensure bronchoscope reprocessing practices adhere to guidelines and manufacturer instructions. The urgency of the current COVID-19 situation underscores the need for robust quality management practices, including audits or virtual audits by qualified experts, visual inspection, and biochemical tests to verify reprocessing effectiveness. These measures are essential for protecting healthcare workers and preventing erroneous BAL test results and bronchoscopy-associated pathogen transmission due to the use of contaminated bronchoscopes.
